# Recurrent Traumatic Brain Injury Surveillance Using Administrative Health Data: A Bayesian Latent Class Analysis

**DOI:** 10.3389/fneur.2021.664631

**Published:** 2021-05-14

**Authors:** Oliver Lasry, Nandini Dendukuri, Judith Marcoux, David L. Buckeridge

**Affiliations:** ^1^Department of Epidemiology, Biostatistics, and Occupational Health, McGill University, Montreal, QC, Canada; ^2^Department of Neurology and Neurosurgery, McGill University, Montreal, QC, Canada

**Keywords:** surveillance, traumatic brain injury, recurrence, recurrent traumatic brain injury, Bayesian analysis, latent class analysis

## Abstract

**Background:** The initial injury burden from incident TBI is significantly amplified by recurrent TBI (rTBI). Unfortunately, research assessing the accuracy to conduct rTBI surveillance is not available. Accurate surveillance information on recurrent injuries is needed to justify the allocation of resources to rTBI prevention and to conduct high quality epidemiological research on interventions that mitigate this injury burden. This study evaluates the accuracy of administrative health data (AHD) surveillance case definitions for rTBI and estimates the 1-year rTBI incidence adjusted for measurement error.

**Methods:** A 25% random sample of AHD for Montreal residents from 2000 to 2014 was used in this study. Four widely used TBI surveillance case definitions, based on the International Classification of Disease and on radiological exams of the head, were applied to ascertain suspected rTBI cases. Bayesian latent class models were used to estimate the accuracy of each case definition and the 1-year rTBI measurement-error-adjusted incidence without relying on a gold standard rTBI definition that does not exist, across children (<18 years), adults (18-64 years), and elderly (> =65 years).

**Results:** The adjusted 1-year rTBI incidence was 4.48 (95% CrI 3.42, 6.20) per 100 person-years across all age groups, as opposed to a crude estimate of 8.03 (95% CrI 7.86, 8.21) per 100 person-years. Patients with higher severity index TBI had a significantly higher incidence of rTBI compared to patients with lower severity index TBI. The case definition that identified patients undergoing a radiological examination of the head in the context of any traumatic injury was the most sensitive across children [0.46 (95% CrI 0.33, 0.61)], adults [0.79 (95% CrI 0.64, 0.94)], and elderly [0.87 (95% CrI 0.78, 0.95)]. The most specific case definition was the discharge abstract database in children [0.99 (95% CrI 0.99, 1.00)], and emergency room visits claims in adults/elderly [0.99 (95% CrI 0.99, 0.99)]. Median time to rTBI was the shortest in adults (75 days) and the longest in children (120 days).

**Conclusion:** Conducting accurate surveillance and valid epidemiological research for rTBI using AHD is feasible when measurement error is accounted for.

## Background

Traumatic brain injury (TBI) leads to significant disability and economic burden in populations across the globe (1, 2). A broad body of research describes the epidemiology of these injuries and assesses ways to mitigate their associated disability (3, 4). However, the overall injury burden reflects not only incident (first-time) injuries, but also recurrent injuries that have different epidemiological characteristics (5–8).

Within a 1-year period after an index TBI, recurrent TBI (rTBI) affects 5-10% of individuals (9). These recurrent injuries are associated with poorer outcomes, such as an increase in post-concussive symptoms leading to productivity losses and long-term complications such as suicide and Chronic Traumatic Encephalopathy (10–15). Despite the important morbidity related to these injuries, there is a paucity of research on how to monitor these recurrences (9, 16). Feasible and timely approaches to conducting public health surveillance of these recurrent injuries is primordial to understanding the TBI burden and assessing whether interventions destined to mitigate them are effective (1, 17).

Administrative health data are a widely available resource for conducting surveillance (4, 18, 19). Although these data are commonly used for surveillance of TBI, their accuracy for conducting rTBI surveillance has not been assessed (9). In addition, there is no perfect reference standard to diagnosis TBI or rTBI. As such, assessing the accuracy of case definitions that detect these injuries requires methods that circumvent the need to define a perfect reference standard (18). The aims of this study were to estimate the measurement error adjusted rTBI incidence and to assess the accuracy of widely-used surveillance case definitions for administrative health data to identify rTBI up to 1 year after an index TBI in children, adults, and elderly across the full spectrum of injury severity, without relying on a gold standard rTBI definition.

## Methods

### Study Design, Population and Data Sources

We used a prospective cohort design to ascertain cases of rTBI in cohorts of index TBI patients, stratified by index injury severity and age group [children (<18 years), adults (18-64 years), and elderly (> =65 years)]. Index TBI patients were identified using a previous latent class analysis that predicted the probability of individuals having an index TBI based on widely-used TBI case definitions in administrative health data (18).

We used a cohort of residents of the Census Metropolitan Area (CMA) of Montreal from 2000 to 2014 to identify incident and recurrent TBI events. The cohort is dynamic with membership maintained to represent a 25% random sample of the CMA of Montreal population. Administrative health data from the Régie de l'Assurance-Maladie du Québec (RAMQ) were used for the analysis (20, 21). These data have been used previously to conduct population-based studies in TBI (22–24). They include all physician claims and the discharge abstract database (DAD) of hospitalizations for members of the cohort. The physician claims data were coded using the ICD-9 CA standard, whereas the DAD was coded using the ICD-9 from 2000 to 2006 and the ICD-10 from 2007 to 2014. Suspected recurrence was defined using four case definitions that are further described below.

To carry over the uncertainty from our original analysis on index TBI to the present analysis, we predicted 1,000 cohorts of index TBI patients using our original Bayesian latent class model on incident TBI (18). These predicted cohorts of incident TBI patients were based on the case definitions for which patients were positive for during their index TBI (eAppendix 2 and eFigure 1) (18). The cohorts of incident TBI patients were predicted in two severity groupings (“mildest/more severe TBI” and “most severe TBI”). The “mildest” and “more severe” index TBI cases from our original analysis on incident TBI were grouped together as the “mildest/more severe” group, which represent patients that were likely to be treated in the outpatient/emergency room setting. The other cohort of patients consisted of the “most severe” TBI cases that were more likely to require hospitalization for their injury (18). We conducted three separate analyses for children, adults, and the elderly, since each of these age groups have unique TBI epidemiological characteristics (2, 4, 17).

We estimated the incidence of rTBI over a 1-year period, using the person-time contribution of the 1,000 predicted index TBI cohorts until 1-year follow-up, censoring from the cohort, or meeting the case definition of a suspected rTBI surveillance case definition.

### rTBI Surveillance Case Definitions

We applied four widely used ICD-based surveillance case definitions for administrative health data to identify suspected rTBI cases (eAppendix 1) (4, 19, 25). The first two case definitions were based on physician claims with a TBI diagnostic code in the outpatient and emergency department, respectively. We defined a third case definition as any TBI diagnostic code contained in the DAD for hospitalizations (primary or secondary diagnosis). Finally, we defined the 4th case definition as any patient that had a radiological examination of the head [computed tomography (CT) scan of the brain, magnetic resonance imaging of the brain or skull x-ray using RAMQ billing codes 08258, 08259, 08570, 08010, and 08013] while simultaneously having a physician claim for any traumatic event (defined as ICD-9 codes 8XX, 91X, 92X, 93X) within 1 day of each other (21, 26). These four case definitions span the full severity spectrum of rTBI patients, from patients only seeking outpatient care to patients requiring hospitalization.

Patients had 1 of 16 (2^4^ = 16) response patterns of positive case definitions based on whether they met these case definitions at least 7 days after their index TBI and up to 365 days after it. We included this temporal constraint since in our previous analysis on incident cases, we had placed a constraint that all claims had to occur within 7 days of the earliest claim for the combination of case definitions to be attributed to the incident TBI. Therefore, all case definitions that were met within the first 7 days after an incident TBI were assumed to be related to the incident event. In our previous analysis on incident TBI, we excluded prevalent cases of TBI by identifying patients that met our TBI case definitions from 1998 to 2000 and excluding them from the follow-up cohort.

### Statistical Analysis

The methods we used to conduct the analysis are similar to the Bayesian latent class analysis we used to assess the accuracy of the same case definitions for index TBI (18). Briefly, we used Bayesian Latent Class Models (BLCMs) to simultaneously assess the accuracy of the four surveillance case definitions defined above (conceptual diagram shown in eFigure 1). Latent class models are used to probabilistically measure unobservable or indirectly observable variables such as the diagnosis of TBI/rTBI (22, 27, 28). The rTBI case definitions we employed provide clues that certain individuals may have incurred a rTBI. By simultaneously assessing whether patients are positive for one or more of the four case definitions, these models can estimate the accuracy of the cases definitions to identify rTBI. This statistical approach circumvents the need to define a gold standard for the TBI diagnosis, which does not exist (23). The diagnostic definition of TBI and rTBI are based on clinical observations and history of clinical events that are often unavailable at the time of assessment. Patients affected by TBI oftentimes have loss of consciousness and amnesia of the event, may have a decreased level of consciousness or may be intoxicated, which makes the accuracy of the diagnosis inaccurate (23). If we were to assume that any diagnostic definition is a perfect reference standard when it is not, the analysis would provide biased inferences (22). These models provide parameter estimates for rTBI incidence, as well as the sensitivity and specificity of the case definitions under study. By using multiple overlapping sources of administrative health data that provide clues to the diagnosis of rTBI, the model adjusts each of these parameters for the inherent measurement error of each case definition in administrative health data without requiring a perfect reference standard (29). In this analysis, the incident TBI model that we previously published was used to construct cohorts of incident TBI patients that adjusts for measurement error to detect incident TBI in administrative health data (18). The current analysis we are conducting on rTBI adjusts for measurement error for administrative health data to detect rTBI. As such, this analysis carries over all the uncertainty from the initial analysis on incident TBI to the current analysis on rTBI, and simultaneously addresses measurement error in the diagnosis of both incident and recurrent TBI. Such an approach ensures that we provide valid parameter estimates that incorporate all of the uncertainty that exists around incident and rTBI diagnoses in administrative health data.

We used logistic regression within the latent class model described above to model the association between sex and rTBI incidence using a sex covariate in the latent class model. This strategy allowed us to assess the sex-specific incidence across each of the three age groups under study. The posterior distributions of each parameter for the 1,000 analyses for each age group and injury severity were then pooled to ascertain our final estimates. We also estimated the overall rTBI incidence and accuracy measures for the four case definitions across the three age groups by pooling the results, weighted by the size of the index TBI cohort of each age group (eAppendix 2) (9, 18, 30–32).

We used a two-class model in the present rTBI analysis (“no rTBI” and “rTBI”) since we only had sufficient degrees of freedom to conduct such an analysis without having to use informative prior information, which is not available in the literature (18). Briefly, with four case definitions we have up to 2^4^ = 16 case definition response patterns, which leads to 15 degrees of freedom. The response patterns of the four case definitions used, across each age group, is provided in eTable 2. In a two-class model there are nine parameters to estimate (an incidence parameter, a sensitivity parameter for each of the four case definitions, and a specificity parameter for each of the four case definitions). Similarly, in a three-class model there are 13 parameters to estimate. Since we predicted 1,000 cohorts of incident TBI and repeated our latent class analysis 1,000 times, there were iterations where zero observations for more than 3 of the 16 response patterns occurred. We attempted to conduct a three-class model but for several iterations of 1,000 analyses non-convergence of the model was observed. Model convergence was not feasible without providing additional prior information on the sensitivity and specificity of case definitions, which is not available in the literature (more details are provided in eAppendix 2) (9). A Bayesian approach was preferred for this analysis since prior distributions can be used as parameter constraints to help with model convergence throughout each of the 1,000 simulated analyses we conducted where certain case definition response patterns are sparse or not observed (even in a two-class model), as well as to perform sensitivity analyses that confirm the robustness of our results (eAppendix 2 and eFigure 2) (30, 33, 34).

### Model Fit, Selection and Convergence

When using latent class analysis, we assume that the multiple case definitions used in the model are conditionally independent given disease status. We verified model fit, and in turn that this assumption was not violated, by conducting posterior predictive checks that assessed the probability that the observed agreement between pairs of case definitions were greater than their predicted agreement (eAppendix 3 and eTable 1) (22, 35). By ensuring appropriate model fit, we confirmed that the two-class model we used was appropriate to provide inferences on rTBI incidence and the accuracy of the four case definitions we were assessing.

We assessed the crude median time to rTBI using the date of the first positive case definition as the time of recurrent injury. We also estimated an adjusted median time to rTBI which adjusted for the probability that an individual was a true incident TBI and rTBI case (eAppendix 4).

### Model Convergence and Sensitivity Analyses

All analyses were conducted in Just Another Gibbs Sampler (JAGS) called from R. Convergence diagnostics were performed by assessing traceplots and the Gelman-Rubin statistic (<1.1). The parameters were sampled from their posterior distribution using three parallel chains of Markov chain Monte Carlo simulations with 20,000 iterations and a burn-in of 5,000 iterations by specifying the likelihood and prior distributions of each one for the Gibbs sampler. 95% credible intervals and medians were reported from the highest posterior densities. We placed constraints on the incidence parameter, the specificity parameters, and two of the sensitivity parameters (ER claims and discharge abstract database) in the form of non-informative prior information. This was completed to help with model convergence across the 1,000 latent class analyses we conducted for each incident TBI cohort that was predicted and to avoid label switching, which can occur in latent class analysis (32). The reporting of this study adhered to the recommended STARD-BLCM guidelines (36). This study was approved by the Institutional Review Board of McGill University's Faculty of Medicine.

We allowed our constraints (prior information) to vary within plausible ranges to assess whether these priors had an impact on the conclusions of the primary analysis (eAppendix 2 and eFigure 2). Finally, we completed a sensitivity analysis to assess how the crude rTBI incidence would vary if we excluded all suspected rTBI claims in the first 30 days after an incident TBI instead of the 7-day window we used in the main analysis. We completed this sensitivity analysis to assess whether or not many suspected rTBI claims in the first 30 days were in fact follow-ups related to the incident TBI and did not represent true recurrent events. In addition, we completed this analysis to assess whether our adjusted rTBI incidence estimated through our Bayesian latent class approach provided a realistic adjustment.

In eAppendix 5, we have provided a step-by-step approach to adjusting crude rTBI incidence using the sensitivity and specificity parameters for given case definitions used to conduct rTBI surveillance in administrative health data. Using this simple approach, stakeholders in rTBI surveillance and epidemiological research in other jurisdictions can adjust for measurement error in rTBI incidence using the parameter estimates provided in this analysis.

## Results

From 2000 to 2014, there were 7,532 suspected rTBI cases within 1 year of their incident event. The crude 1-year rTBI incidence was 8.03 (95% CrI 7.86, 8.21) per 100 person-years. The measurement error-adjusted rTBI incidence from the Bayesian latent class analysis was lower [4.48 (95% CrI 3.42, 6.20) per 100 person-years]. The adjusted median time to recurrence was more delayed across all age groups than the crude estimate (Table 1). There were significant differences in the median time to rTBI by age group, with adults having the shortest median time (75 days) to rTBI and children the longest (120 days). Patients with “most severe” incident TBI had a shorter adjusted median time to rTBI compared to patients with “mildest/more severe” incident TBI across all three age groups (eTable 3).

**Table 1:** Summary of suspected incident TBI and rTBI cases identified from administrative health data surveillance case definitions for the Montreal census metropolitan area population from 2000 to 2014.

	**“Suspected” incident cases (*n*)**	**Mean predicted cohort size of “true” incident TBI (*n*, across 1,000 simulations)**	**“Suspected” recurrent cases (*n*)**	**rTBI crude incidence per 100 person-years (95% CrI)**	**Crude median time to recurrence (days)**	**Adjusted median time to recurrence (days) (95% CrI)**
**Children (0-17)**	30,433	35,161 Male: 60.3%“ Most severe”: 7.82%	1,567 [1,607]^*^	5.2 (4.9, 5.4)	98	120 (116, 125)
Outpatient claim	7,992		600			
Emergency room claim	20,119		847			
Hospital physician claim	877		[92]^*^			
Discharge abstract database	1,657		62			
Radiological exam	5,029		234			
**Adults (18-64)**	38,486	38,454 Male: 56.7% “Most severe”: 11.1%	3,205 [3,414]^*^	8.3 (8.1, 8.6)	25	75 (73, 77)
Outpatient claim	8,710		1,489			
Emergency room claim	16,825		646			
Hospital physician claim	1,911		[637]^*^			
Discharge abstract database	2,697		338			
Radiological exam	19,243		1,013			
**Elderly (65+)**	24,881	23,655 Male: 37.9% “Most severe”: 10.0%	2,760 [3,193]^*^	11.1 (10.7, 11.5)	39	109 (108, 110)
Outpatient claim	1,873		274			
Emergency room claim	7,427		539			
Hospital physician claim	1,725		[934]^*^			
Discharge abstract database	2,934		544			
Radiological exam	18,635		1,884			

*Distribution of patients that were positive for at least 1 of the case definitions for rTBI in administrative health data across the three age groups in the study. The estimates of mean predicted cohort size and adjusted median time to recurrence were calculated as described in eAppendices 2 and 4, respectively. The proportion of males in the cohort and the proportion of patients that were in the “most severe” stratification of the predicted cohorts is also provided*.

* *The hospital (inpatient) physician claims were not used in the rTBI analysis but were used in the incident TBI analysis. In brackets, the total suspected rTBI cases are shown when the hospital physician claims are included to identify rTBI cases. CrI, credible interval*.

The rTBI incidence was most elevated for the elderly population (9.03 per 100 person-years, 95% CrI 7.68, 10.24) and the lowest in children (1.69 per 100 person-years, 95% CrI 1.11, 2.73). When comparing male and female incidence across the entire population of incident TBI patients, female sex was associated with a higher incidence of rTBI. However, male sex was associated with a higher incidence of rTBI compared to female sex when individually assessing the incidence of rTBI in children and adults. In addition, the incidence of rTBI between males and females was similar in the elderly. Patients with a “most severe” index TBI had a significantly higher incidence of rTBI in comparison to patients with a “mildest/more severe” index TBI, across all age groups (Table 2).

**Table 2:** Measurement error-adjusted rTBI incidence by age group for the Montreal census metropolitan area population from 2000 to 2014.

	**rTBI incidence per 100 person-years (95% CrI)**	**M:F**	**S:M**
Children (0-17 years)	1.69 (1.11, 2.73)	1.142 (0.918, 1.453)	1.685 (0.432, 3.807)
Adults (18-64 years)	3.57 (2.39, 5.16)	1.409 (1.130, 1.673)	3.219 (1.853, 4.626)
Elderly (65+ years)	9.03 (7.68, 10.24)	0.999 (0.920, 1.100)	1.051 (0.852, 1.313)
Across all age groups	4.48 (3.42, 6.20)	0.857 (0.737, 1.024)	1.824 (1.146, 2.478)

*The measurement error-adjusted incidence of rTBI across age groups and index TBI severities are as shown above. The male:female (M:F) and “Most severe”:“mild/more severe” (S:M) index TBI severity incidence ratios are also shown. CrI, credible interval*.

**Figure 1:**
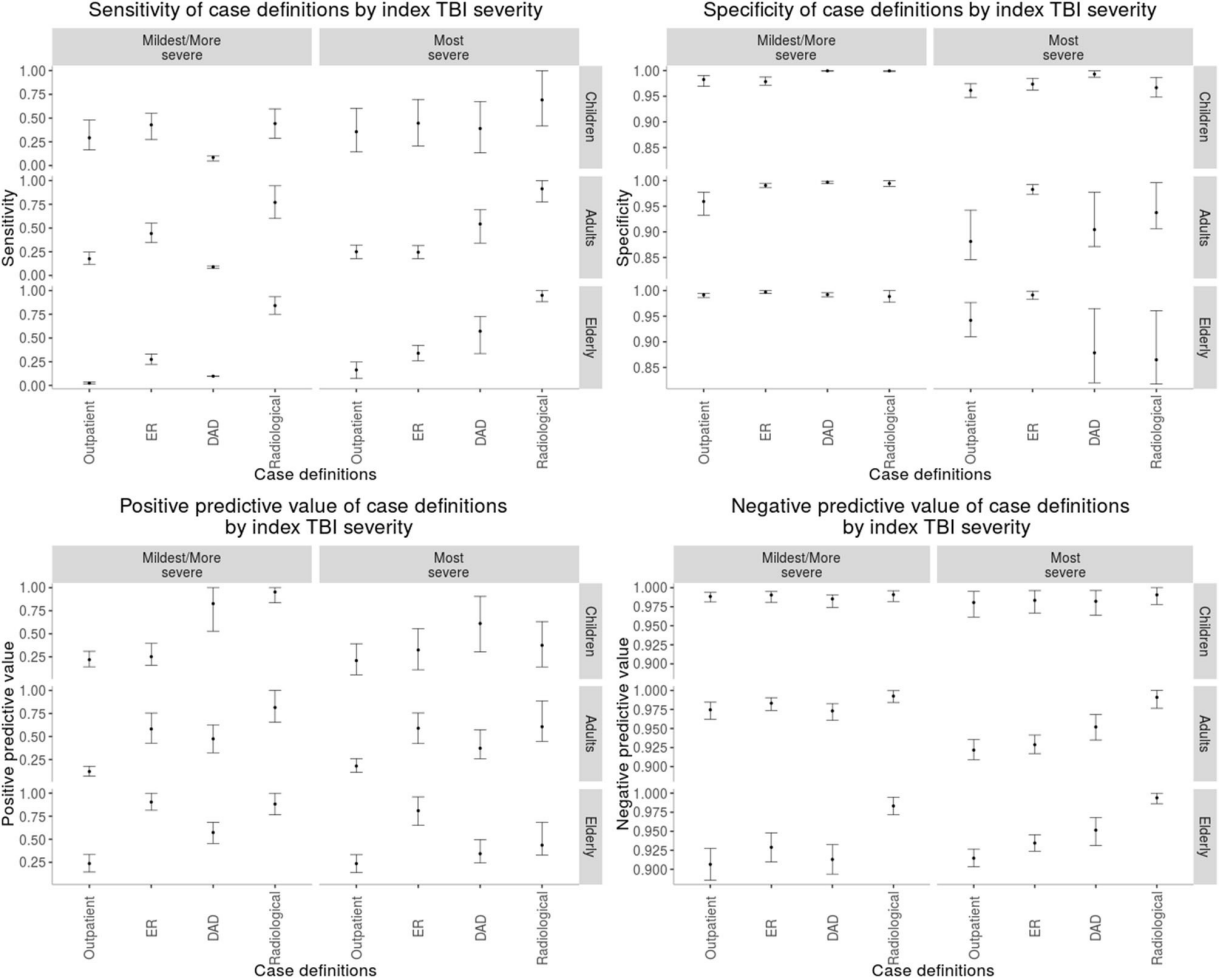
Performance of each case definition to perform rTBI surveillance stratified by each age group and incident TBI severity. “Outpatient,” outpatient claims; “DAD,” discharge abstract database; “Radiological,” radiological examination claim in the context of any trauma diagnosis.

The most sensitive rTBI case definition was based on a radiological examination with a diagnosis of trauma and the least sensitive was based on the DAD, except for the elderly where the outpatient claims were the least sensitive. In children, the case definition based on the DAD was the most specific, whereas in adults and the elderly it was the emergency room physician claim. The least specific case definition in children was the ER claim. The DAD and radiological examination case definition had the highest PPV in children. In contrast, in adults the radiological examination and in the elderly the ER claim had the highest PPV, respectively (Table 3). As shown in Figure 1, there was heterogeneity in the performance of case definitions across age groups and index TBI severity.

**Table 3:** Performance of surveillance case definitions to detect rTBI cases in administrative health data stratified by age group for the Montreal census metropolitan area population from 2000 to 2014.

	**Sensitivity (95% CrI)**	**Specificity (x10^**−2**^)(95% CrI)**	**Positive predictive value (95% CrI)**	**Negative predictive value (95% CrI)**
**Children**				
Outpatient claim	0.29 (0.18, 0.45)	9.817 (9.688, 9.888)	0.21 (0.15, 0.29)	0.988 (0.981, 0.992)
ER claim	0.44 (0.31, 0.55)	9.774 (9.720, 9.853)	0.25 (0.17, 0.37)	0.990 (0.982, 0.994)
Discharge abstract database	0.11 (0.07, 0.15)	9.992 (9.977, 9.999)	0.74 (0.51, 0.93)	0.985 (0.976, 0.990)
Radiological examination of head with a diagnosis of trauma	0.46 (0.33, 0.61)	9.973 (9.920, 9.999)	0.77 (0.58, 0.89)	0.991 (0.984, 0.996)
**Adults**				
Outpatient claim	0.19 (0.13, 0.25)	9.516 (9.164, 9.694)	0.13 (0.08, 0.18)	0.969 (0.955, 0.980)
ER claim	0.42 (0.33, 0.52)	9.898 (9.851, 9.939)	0.60 (0.48, 0.74)	0.979 (0.967, 0.987)
Discharge abstract database	0.14 (0.11, 0.21)	9.876 (9.726, 9.948)	0.31 (0.21, 0.44)	0.969 (0.956, 0.979)
Radiological examination of head with a diagnosis of trauma	0.79 (0.64, 0.94)	9.874 (9.762, 9.963)	0.70 (0.55, 0.85)	0.992 (0.983, 0.999)
**Elderly**				
Outpatient claim	0.04 (0.03, 0.05)	9.872 (9.814, 9.910)	0.24 (0.17, 0.31)	0.912 (0.900, 0.925)
ER claim	0.29 (0.25, 0.35)	9.957 (9.928, 9.988)	0.87 (0.79, 0.96)	0.934 (0.922, 0.947)
Discharge abstract database	0.15 (0.12, 0.18)	9.809 (9.746, 9.874)	0.44 (0.35, 0.53)	0.921 (0.910, 0.933)
Radiological examination of head with a diagnosis of trauma	0.87 (0.78, 0.95)	9.732 (9.602, 9.866)	0.76 (0.66, 0.87)	0.987 (0.977, 0.996)

*Overall performance of each of the four case definitions for each age group across both index TBI severities. CrI, credible interval*.

Model fit across the three age groups was deemed appropriate based on posterior predictive checks and sensitivity analyses where prior information was varied within plausible ranges (eTable 1 and eFigure 2). More specifically, our sensitivity analyses tested the impact of several assumptions on the prior information we used. Across the sensitivity, specificity, and incidence parameters for all age groups, the sensitivity analysis estimates demonstrated 95% credible intervals that overlapped with our main analysis findings, indicating that the overall conclusions of our study were robust to multiple assumptions on the prior information we used.

Crude rTBI incidence decreased across all three age groups when we excluded all suspected rTBI claims in the first 30 days after the incident TBI (eTable 4). For each age group, this modified crude rTBI incidence approached the adjusted rTBI incidence we estimated using the Bayesian latent class models we developed.

## Discussion

The impact of rTBI on the overall TBI burden has been overlooked in the general population (9, 16). This study provides the first assessment of the accuracy of administrative health data to conduct rTBI surveillance across the full severity spectrum of injuries, without relying on a gold standard TBI/rTBI definition (9). The performance of surveillance case definitions and incidence estimates vary significantly by age groups and index TBI severities. We have demonstrated that accurate rTBI surveillance is feasible using widely applicable surveillance case definitions in administrative health data, when measurement error is accounted for.

### Accuracy of Surveillance Case Definitions to Conduct rTBI Surveillance and Epidemiological Research

There has been a considerable amount of research on the methodology used to conduct incident TBI surveillance (1, 3, 18, 25, 37). However, a systematic review of the epidemiology of rTBI in the general population demonstrated the lack of similar research for rTBI (9). The present study demonstrates that the accuracy of case definitions to detect rTBI is different from the accuracy of cases definitions to detect incident TBI. This finding may be explained by the different patterns of care patients follow for an rTBI compared to an index TBI. For example, our previous study demonstrated that the case definition using radiological examination claims with a concomitant diagnosis of any trauma had the highest sensitivity for TBI in adults and the elderly (18). This case definition had a lower sensitivity for TBI in children, probably due to the concern of radiation exposure and its consequences (38, 39). In contrast, the present study demonstrates that the radiological examination case definition is more sensitive for rTBI than for incident TBI across all age groups. More specifically, this case definition's sensitivity in children was 14% for incident TBI vs. 46% for rTBI, in adults was 48% for incident TBI vs. 79% for rTBI, and in the elderly was 66% for incident TBI vs. 87% for rTBI (18). Radiological examinations may be more sensitive for rTBI as compared to incident TBI, including for children, because clinicians may be using imaging studies more liberally for patients with rTBI; these patients may have a more severe clinical presentation and be at risk of more severe complications related to repeated head trauma (16, 40). In addition, guidelines to conduct imaging in the context of TBI have only been validated for incident TBI, which may lead to more widespread use of imaging in rTBI since there are no guidelines to limit its use (26, 41).

### rTBI Incidence

A wide variation in the estimates of rTBI incidence in the general population has been reported, due to heterogenous surveillance methods used (9). Comparing the 1-year incidence of rTBI occurrence, studies published in the literature reported a range of estimates from 5.5-10% (9). Two of these studies focused on pediatric populations (42, 43). Our estimate of rTBI incidence in children was lower (1.69 per 100 person-years, 95% CrI 1.11, 2.73). This result is likely explained by differences in methodology. First, in these two studies, parent self-report was used as the outcome for rTBI, which may overestimate the true incidence. In contrast, our crude incidence of rTBI in children was 5.20 per 100 person-years in children, which is similar to the estimates published by Swaine et al., which used the Quebec pediatric population as we did (42). In both of our studies, only single rTBI events were included, and multiple events were not accounted for. In their study, parents provided a self-report of children requiring medical care for a rTBI, which would help limit the overestimation of rTBI incidence. However, we would expect our rTBI incidence estimates to be lower than the incidence they report because of the difference in patient populations that we studied. In their study, they assessed the risk of rTBI in a cohort of children with TBI that were assessed at pediatric neurotrauma centers, whereas the children in our cohort sought care at both neurotrauma and community hospitals. As such, the TBI severity of our cohort is expected to be lower than their cohort. Since higher incident TBI severity is associated with higher rTBI incidence, we would expect our rTBI incidence to be lower. Without measurement-error adjustment, we would have overestimated the rTBI incidence in our cohort of children with milder incident TBI. As such, measurement error adjustment is necessary to accurately conduct rTBI surveillance using administrative health data.

The rTBI incidence we reported in adults and the elderly was higher than in children, which is in keeping with previous studies demonstrating that increasing age is a risk factor for rTBI (44, 45). Theadom et al. completed an assessment on the rTBI incidence in the general population in New Zealand (16). They reported a rTBI incidence of 9.9% at 1-year follow-up, in comparison to 4.48 (95% CrI 3.42, 6.20) per 100 person-years in our present study. As mentioned above, only a single rTBI event was included as part of the analysis in Theadom et al.'s study as well as ours, and multiple events were not estimated. The difference in estimates can be explained by many factors. First, Theadom et al.'s study used a cohort study design to ascertain all cases of incident TBI in two defined regions of New Zealand, and then assessed rTBI up to 1 year after the index injury. However, only 52% of eligible incident TBI cases were included in the follow-up for their assessment of rTBI risk, which may have biased the results. Many baseline covariates compared between participants and non-participants were similar. Nonetheless, injury severity was not compared between these two groups. For example, if many milder cases, compared to more severe cases, were to preferentially not participate, there may be an overestimate of rTBI risk, since more severe index injuries have a higher incidence of recurrence. Also, Theadom et al. were able to identify rTBI who did not present to medical care, which we were unable to assess. As above, measurement error may have also contributed to an overestimate of their reported 1-year risk.

### Variation of rTBI Incidence and Case Definition Accuracy Across Age, Sex, and Index TBI Severity

As for index TBI, the incidence of rTBI varies by age and sex. The incidence of recurrence increases with increasing age (44, 45). In comparison to the index TBI, rTBI does not seem to have a bimodal peak among children and the elderly (46). Male patients tend to have a higher incidence of rTBI compared to females in both children and adults. However, the incidence of rTBI in elderly females and males was similar. A systematic review assessing the association between sex and rTBI found no conclusive evidence supporting this association, with many studies reporting unprecise association measures crossing the null (9). When assessing the association of sex and rTBI incidence across the entire population, we demonstrated that female sex was associated with a higher risk of rTBI. The reason for this finding is that the elderly population had a significantly higher incidence of rTBI compared to adults and children. In addition, the proportion of females in the elderly population is higher than in children and adults. For these reasons, female sex appears to be associated with a higher risk of rTBI when assessing the association across all age groups, but when we completed our stratified analyses by age group, we demonstrated that female sex was actually associated with a lower incidence of rTBI in children and adults. In short, the association between sex and rTBI incidence across the entire population is confounded by age in our study.

We also observed that the adjusted rTBI incidence for children and adults was significantly different than the crude rTBI incidence in these age groups. However, in the elderly the crude rTBI and adjusted rTBI incidence were similar. Although we cannot conclude why the latter was observed, one hypothesis is that elderly patients are more likely to be frail and require longer hospitalizations or rehabilitation after an incident TBI. As such, early claims after an incident TBI may not occur since these patients do not have follow-up appointments recorded in administrative health data while they are hospitalized. Thus, false rTBI claims that are actually follow-ups are less likely in the elderly population. Our sensitivity analysis where we restrict our analysis to use TBI claims 30 days after an initial TBI supports this argument, since the crude rTBI incidence was the least reduced for the elderly cohort when completing this sensitivity analysis.

Our study emphasized that index TBI severity is an important determinant of rTBI. Patients with a “most severe” index TBI had a significantly higher incidence of a recurrent injury compared to patients with milder injuries. However, the precision of estimates varied widely across the three age groups. Previous studies have reported similar findings, although the magnitude of association was smaller than in the present study (44, 45, 47, 48). This discrepancy can be explained by the fact that our study included patients across the entire spectrum of injury severity, whereas other studies have tended to include only hospitalized patients or only patients presenting to the emergency department (9). We also compared the rTBI incidence of the “most severe” index TBI cases (patients likely to be hospitalized) to those with a “mildest/more severe” index TBI (patients unlikely to be hospitalized). As such, we contrasted groups with a greater difference in severity in comparison to previous studies. Interestingly, this observation raises the possibility that there exists a dose-response relationship between index TBI severity and rTBI incidence; as the severity of the index TBI increases, the incidence of rTBI appears to increase (49).

The performance of the surveillance case definitions also varied by index TBI severity. We demonstrated that among the cohort of patients with a “mildest/more severe” index TBI, sensitivity was highest for case definitions based on emergency department physician claims and radiological examinations. In contrast, for patients with a “most severe” index TBI, the DAD and radiological examinations case definitions tended to have the highest sensitivity. Moreover, the specificity and PPV of the DAD and radiological examinations case definitions were higher in patients with a “mildest/more severe” index TBI compared to a “most severe” index TBI. A possible explanation for these findings is that patients with a higher severity index TBI are more likely to obtain follow-up imaging for their index injury and may be readmitted to a rehabilitation center where a new DAD entry is entered, which leads to more false positives. In short, the variability in how the case definitions perform by age group and index TBI severity is important for stakeholders in surveillance and epidemiological research who may be investigating the incidence of rTBI in specific TBI subpopulations.

### Time to rTBI and Opportunities for Prevention

Assessing the time-to-recurrence of rTBI is important as it defines a window of opportunity during which interventions may help mitigate the risk of rTBI. We identified that the median time to recurrence varied from 75 to 120 days, depending on the age group, which is in keeping with previous research (16). Our study additionally demonstrated that patients with higher severity index TBI also have a shorter median time to rTBI compared to patients with milder injuries. Although we cannot confirm the underlying reason for this finding, we can hypothesize that patients with higher severity index TBI may have a higher propensity to have a TBI due to behavioral or environmental factors, such as a risk for falls or participation in certain recreational activities. Our administrative health data did not provide the external causes of injury (mechanisms of injury) for patients and as such we cannot confirm these findings. However, further research on the topic is warranted so that high-risk groups for early rTBI can be identified. Nonetheless, interventions that mitigate the occurrence of rTBI must be implemented soon after an index case regardless of the severity of the incident TBI. Unfortunately, there is no published evidence describing interventions that may reduce the incidence of rTBI (9).

Clearly, more research is necessary to identify strategies to reduce the risk of rTBI, which tends to occur within the first few months post-index injury. The accuracy of case definitions to identify rTBI is important to consider when conducting epidemiological research. By using the methods and results from this study, investigators have the tools to construct cohorts of index TBI patients, assess their outcome of rTBI accurately, and thereafter make valid inferences regarding the association between interventions and rTBI. For example, preliminary research on the impact of treating patients in a specialized neurotrauma center as opposed to a non-specialized center, for equally severe injuries, is associated with a lower risk of rTBI (50). Further research on such health care or public health interventions that may mitigate the rTBI burden in the general population is required.

### Limitations

Our study has limitations that should be considered. First, we used administrative health data from a single jurisdiction, which may limit the generalizability of the results across other health regions. Nonetheless, administrative data tend to be similar across jurisdictions and extensive research on TBI epidemiology demonstrates that TBI epidemiological characteristics are consistent across the developed word (2). Second, we used prior information, which assisted with model convergence, but may have influenced our results. However, we used plausible priors and completed several sensitivity analyses to demonstrate the robustness of our main analysis' results. Third, our study only includes patients that sought medical care for their index TBI and rTBI. As such, our results likely represent an underestimate of the true injury burden. Fourth, when using administrative health data claims, follow-up visits, rehospitalizations for other causes, and follow-up radiological examinations for an incident TBI may be falsely classified as rTBI events. Our latent class analysis circumvented this problem by using overlapping administrative health data with information provided by different providers. In addition, we completed a sensitivity analysis where we excluded suspected rTBI claims in the first 30 days after an incident TBI to exclude claims that may be related to the incident TBI and not a recurrence. This sensitivity analysis demonstrated that the crude incidence decreases toward the adjusted estimate we obtained using our Bayesian analysis that adjusts for this type of measurement error. As such, this sensitivity analysis supports the methodology we used to adjust for measurement error. We must emphasize that sensitivity and specificity parameters we are reporting are representative of how the case definitions perform when excluding suspected TBI claims for the first 7 days after an incident TBI. These parameters would perform differently if different time-period windows were used to exclude suspected TBI claims. Fifth, we were only able to assess single rTBI events that occurred over a 1-year period after an incident TBI. Clearly, multiple recurrent events may occur and significantly increase the overall TBI burden. Using our latent class approach where we used administrative health data claims to elucidate patterns of health care utilization of suspected rTBI cases, we were unable to discriminate whether claims were related to a first recurrence or a subsequent recurrence. As such, our estimates represent the rTBI burden of single recurrences, but the true burden of rTBI is likely larger. This phenomenon should be further investigated in future studies. Sixth, our results depend on assumptions related to the latent classes we used in our incident and rTBI analyses. For the incident TBI analysis, we had three latent classes (“mildest,” “more severe,” and “most severe” TBI). In our rTBI analysis we were only able to use two latent classes because of a lack of sample size to complete an analysis with more latent classes, without having to use more prior information that is not available in the literature. As such we are assuming that incident TBI and rTBI patients are appropriately labeled by the classes we have created, but further research that compares clinical data of these patients, such as through chart reviews, would be necessary to confirm that patients were appropriately labeled into latent classes. Nonetheless, we completed numerous sensitivity analyses in both our prior study on incident TBI and the present study on rTBI; prior information was varied and model fit was assessed to ensure that our models had an appropriate fit and interpretation of the data (18). Lastly, we needed to group our analysis in three large age groups because we did not have sufficient power in our study to complete a more granular analysis of rTBI incidence by smaller age groups without using additional prior information which was not available in the literature. Future studies with larger populations should consider assessing age-specific rTBI incidence in a more granular fashion.

## Conclusion

Recurrent TBI is an important contributor to the overall population burden of TBI. Administrative health data can be used to conduct accurate and efficient rTBI surveillance by adjusting for measurement error. The methods and results from this study provide stakeholders in rTBI with tools and information to justify the allocation of resources toward prevention and care for rTBI. They can also inform and enable epidemiological research that investigates strategies to reduce the rTBI burden and thereby help address the overall TBI burden.

## Data Availability Statement

The data analyzed in this study is subject to the following licenses/restrictions: The dataset used are Quebec's (RAMQ) administrative health data that cannot be publicly shared. Requests to access these datasets should be directed to Oliver Lasry, oliver.lasry@mcgill.ca.

## Ethics Statement

This study was reviewed and approved by the Institutional Review Board of McGill University's Faculty of Medicine. Written informed consent from the participants' legal guardian/next of kin was not required to participate in this study in accordance with the national legislation and the institutional requirements.

## Author Contributions

OL devised the research questions, conceptualized the study design, planned and executed the statistical analysis, interpreted the results, and drafted the final manuscript. ND helped design the statistical plan, reviewed the analysis, and critically reviewed the final manuscript. JM helped with designing the study and establishing the case definitions used, in addition to critically reviewing the final manuscript. DB helped with designing the study, reviewing the statistical plan, and critically reviewed the final manuscript. All authors contributed to the article and approved the submitted version.

## Conflict of Interest

The authors declare that the research was conducted in the absence of any commercial or financial relationships that could be construed as a potential conflict of interest.
