# Supplementary material for: Recurrent Traumatic Brain Injury Surveillance Using Administrative Health Data: A Bayesian Latent Class Analysis
Source: Front Neurol. 2021 May 14;12:664631. doi: 10.3389/fneur.2021.664631 (PMC8160293; doi:10.3389/fneur.2021.664631)
Supplement: Supplementary file 1 [file Data_Sheet_1.docx]

###

### Supplementary Digital Content

**eAppendix 1: International classification of disease codes used to ascertain traumatic brain injury cases from 2000 to 2014**

| **Case definition** | **ICD-9** | **ICD-10*** | **RAMQ procedure code** |
| --- | --- | --- | --- |
| **Outpatient physician claim** | 800.X  801.X  803.X  850.X-854.X  950.1  950.2  950.3  959.0 | N/A | N/A |
| **Emergency department claim** | 800.X  801.X  803.X  850.X-854.X  950.1  950.2  950.3  959.0 | N/A | N/A |
| **Inpatient physician claim** | 800.X  801.X  803.X  850.X-854.X  950.1  950.2  950.3  959.0 | N/A | N/A |
| **Hospitalization discharge abstract database** | 800.X  801.X  803.X  850.X-854.X  950.1  950.2  950.3  959.0 | S01.X  S02.1  S02.3  S02.7  S02.8  S02.9  S04.X  S06.X  S07.X  S09.7  S09.8  S09.9  T02.0  T02.10  T04.0  T06.0  T90.X | N/A |
| **Radiological exam of the head within 1 day of a claim for any trauma diagnosis** | 8XX  91X  92X  93X  94X  95X | N/A | 08258  08259  08570  08010  08013 |

ICD codes used in the main analysis to identify recurrent traumatic brain injury. The ICD-9 codes are taken from the surveillance definition developed by the Centers for Disease Control, while the ICD-10 codes are taken from a systematic review identifying the commonly used ICD-10 case definitions for traumatic brain injury.^1,2^ *The hospitalization discharge abstract case definition made use of the ICD-9 iteration from 2000-2005 and the ICD-10 iteration from 2006-2015. The radiological exam of the head case definition was defined as patients that had a head imaging claim by a radiologist within 1 day of having any trauma diagnosis claim by another physician.^3^ ICD = International classification of disease, RAMQ = Régie de l’assurance maladie du Québec

**eAppendix 2: Model specification of the latent class model**

Simulated cohorts of incident TBI cases by age group and index TBI severity:

The analysis used *j*=1000 predicted cohorts of incident TBI cases across all three age groups. The probability of an individual having an incident TBI of specific severity *Sev* given a response pattern of positive case definitions $T_{i}$=1…32 was used to predict cohorts of incident TBI. 32 response patterns were available given that *p*=5 case definitions were used in the incident TBI analysis (2^5^=32 patterns). These probabilities $P\left( Sev=s|T_{i} \right)$ were ascertained from our analysis that investigated incident TBI in the same population with the same case definitions.^4^ The posterior predictive distribution from this previous analysis was sampled 1000 times to ascertain the 1000 predictions. Patients not positive for any case definition in the index TBI analysis also had a small non-zero probability to have a diagnosis of TBI. By definition, these individuals could not be positive for any suspected rTBI since they were not positive for any case definition. Thus, a follow-up time after incident TBI could not be defined for these individuals. We assumed they were followed for 365 days since the average follow-up time in the cohort we used is greater than 365 days.

The predicted cohorts were stratified by incident injury severity into two groups: “mildest/more severe” and “most severe” TBI, as was described in the previous analysis on incident TBI. We were unable to stratify across the 3 injury severities we had identified in our previous latent class analysis due to a lack of power, which led to non-convergence of the latent class model. We therefore decided to group the “mildest” and “more severe” groups together, since they represent a group of incident TBI patients that are primarily managed in the outpatient setting, whereas the “most severe” group represents patients that are hospitalized and receive more advanced care. We stratified the analysis to allow all accuracy parameters and incidence parameters to vary by index TBI severity. As such, two cohorts for each age group were predicted for a total of 6 predicted cohorts that were subsequently each analyzed using the latent class model described below. Using the aforementioned probabilities, a categorical distribution with the probability distributions were used to classify individuals into one of three categories of incident TBI: “no TBI”, “mildest/more severe TBI”, or “most severe TBI”. The latter was repeated *j*=1000 times and the category that an individual was placed in, ${Sev}_{ij}$, was based on their pattern of positivity for the 5 case definitions. As such, a “mildest/more severe” (*Sev* = *s* = 2) and a “most severe” (*Sev* = *s* = 3) cohort was formed 1000 times for each age group. The latent class analysis described below was repeated 1000 times for each of these two severity groups of incident TBI and for the three age groups. The parameter estimates from each of the 1000 simulations were pooled together to get final overall summary estimates across all simulated cohorts from the posterior distribution of each analysis. By simulating these 1000 cohorts for each incident TBI severity and age group, we ensured that all the uncertainty of the incident TBI diagnosis from the initial analysis was carried over to the present analysis on rTBI. eFigure 1A and 1B provide heuristic diagrams that describe the latent class models that were used to predict the cohorts and to complete the rTBI analysis, respectively.

For the incident TBI cohort predictions for the “mild/more severe TBI group” - *s*=2:

${Sev}_{ij}\sim categorical(P\left( Sev=s|T_{i} \right))$ for *j*=1:1000

$${Sev}_{ij}=1 if {Sev}_{ij}=2$$

${Sev}_{ij}=0 if {Sev}_{ij}=1$

For the incident TBI cohort predictions cohort for the “most severe incident TBI group” - *s*=3:

${Sev}_{ij}\sim categorical(P\left( Sev=s|T_{i} \right))$ for *j*=1:1000

$${Sev}_{ij}=1 if {Sev}_{ij}=3$$

${Sev}_{ij}=0 if {Sev}_{ij}=1$

N.B.: The incident TBI cohort predictions were completed independently for *s*=2 and *s*=3 in each age group as shown directly above.

$n_{ij,Sev=s}=$ ${Sev}_{ij}*n_{i}$ for *s*=2,3

Where $n_{i}$ are the counts of individuals with an *i*^th^ case definition response pattern in the incident TBI cohort.

$N_{j,Sev=s}= \sum_{i=1}^{32} n_{ij,Sev=s}$ for *s*=2,3 where $N_{j,Sev=s}$ is the total size of the incident TBI cohort for the *j*^th^ simulation and *Sev*=*s* severity (for *s*=2,3).

By forming these cohorts of individuals with “true” incident TBI, we were able to follow individuals for rTBI case definitions after their incident TBI for each of the *j*^th^ simulations. As shown above, $n_{ij,Sev=s}$ can be a positive integer or 0, depending on whether a given simulation categorized an *i*^th^ case definition response pattern to be a “true” incident TBI or not. In the case where $n_{ij,Sev=s}$ was 0, individuals with this incident TBI case definition response pattern were not considered to have TBI and therefore were not followed for the rTBI case definition response patterns described below. In addition, their person-time contribution to the analysis was not considered since they were not considered as part of the cohort for that particular *j*^th^ prediction. More specifically, six groups of incident TBI cohorts were predicted for each severity *s*=2 and *s*=3 within each age group (children, adults, and elderly).

*p=1* = outpatient physician claims

*p=2* = emergency room claims

*p=3* = inpatient claims

*p=4* = TBI diagnosis in discharge abstract database

*p=5* = radiological examination of the head in the context of any other traumatic injury

*s=1* = Incident class 1 (“No TBI”)

*s=2* = Incident class 2 (“Mildest/more severe TBI”)

*s=3* = Incident class 3 (“Most severe TBI”)

Bayesian latent class model

The basic latent class model we used in the analysis is shown below. In short, there are 16 possible responses to the *pr*=4 case definitions (2*^pr^* = 2^4^ = 16). We excluded inpatient physician claims with a diagnosis of TBI since many of these claims represent daily claims that physicians submit during prolonged hospitalizations for an index TBI. The other case definitions we used circumvent this problem since the patient must leave the hospitalization setting to be positive for another case definition. In addition, the DAD provides the same information regarding inpatient hospitalizations for TBI. Also, strong correlation between these two case definitions was identified when we attempted an analysis where inpatient physician claims were used. As such, these claims were excluded to maintain model parsimony and avoid bias through correlation with the DAD case definition (see explanation below on conditional independence of case definitions).

Since our analysis allows rTBI incidence to vary by sex, the number of responses is actually 32 since there are 16 possible responses for each sex. The latent class analysis models the probability of having a response vector $T_{irj,Sev=s}$= 1…16 of the *pr* = 1…4 case definitions used in the model for 1 year after occurrence of an incident TBI for each *j*^th^ simulated cohort (a total of 32 response vectors) with *Sev*=*s* (where *s*=2 or *s*=3). The person-years ($n_{irj,Sev=s}$) contributed over a one-year period after an incident TBI to each combination of responses is modelled with a multinomial distribution where $N_{rj, Sev=s}$ (for *j*=1000) is the total count of person-years contributed after incident TBI in the *j*^th^ simulated incident TBI cohort. The accuracy parameters are the sensitivity of the *pr^th^* case definition – $P\left( T_{iprj} \right|L_{ikrj}$). The incidence of rTBI, ${P(L}_{ikrj})$, is allowed to vary by sex through a logistic regression model shown below. The ${\alpha\_male}_{kr}$ and ${\beta\_female}_{kr}$ parameters are heterogeneity variables that allow the incidence to vary by sex (${sex}_{ij,Sev=s}$). The “*r*” subscript indicates that the parameter in the model represents the rTBI analysis, in contrast to the incident TBI analysis from our previously completed investigation on the topic. The “*Sev*” subscript indicates the severity cohort under analysis. Six latent class models were conducted; one for each age group and index TBI severity (3 age groups with 2 index TBI severity cohorts per age group provides a total of 6 analyses). The posterior distribution of each parameter for each simulation was estimated using Gibbs sampling of the full conditional distributions defined below.^5^ These posterior distributions for each parameter/age group/severity were pooled together to ascertain our final estimates that retain all uncertainty from our analyses.

*pr=1* = outpatient physician claims

*pr=2* = emergency room claims

*pr=3* = TBI diagnosis in discharge abstract database

*pr=4* = radiological examination of the head in the context of any other traumatic injury

*kr=1* = Recurrent class 1 (No rTBI)

*kr=2* = Recurrent class 2 (rTBI)

General latent class model and likelihood:

$P\left( T_{irj,Sev=s} \right)=\sum_{kr=1}^{Kr} P(L_{ikrj,Sev=s})\prod_{pr=1}^{Pr} P\left( T_{iprj,Sev=s} \right|L_{ikrj,Sev=s})$

$$Likelihood \propto\prod_{i=1}^{32} P\left( T_{irj,Sev=s} \right)$$

$$n_{irj,Sev=s}|T_{irj,Sev=s},N_{rj,Sev=s}\sim multinomial (P(T_{irj,Sev=s}), N_{rj, Sev=s})$$

Where $n_{irj}$and $N_{rj}$ is predicted for each severity (*s*=2 and *s*=3) and age group from the *j*=1000 simulated cohorts described above.

Logistic regression to model variability in incidence by sex:

$logit(P\left( L_{ikrj,Sev=s} \right)= {\alpha_{male}}_{krj,Sev=s}+ {\beta_{female}}_{krj,Sev=s}*{sex}_{ij,Sev=s} (for k= 2)$

$$P\left( L_{i1rj,Sev=s} \right)=1-P\left( L_{i2rj,Sev=s} \right)$$

Constraints and assumptions using prior information:

Since label switching can lead to non-convergence of latent class models, we imposed constraints in the form of relatively non-informative prior distributions, which were informed by previous literature.^6^ In our 2-class model for the “mild/more severe” index TBI cohort analysis, we forced discharge abstract database to have a maximum sensitivity of 10% and for the emergency room physician claim to have a sensitivity of at least 10%. In addition, we constrained the specificity of all 4 case definitions to be at least 80%, which is quite uninformative based on previous literature on index TBI surveillance.^4^ For the “most severe” index TBI cohort, we only required constraints on the specificities. These constraints were used to allow the model to converge. They are considered relatively uninformative priors given that the literature has shown that less than 5% of mild TBI patients that have rTBI are admitted to hospital.^7^ Our previous analysis on incident TBI also found that the sensitivity of case definitions for index TBI are well within the range of these constraints.^4^ We also constrained the incidence in males to 10% since the highest 1-year risk of rTBI was reported to be 9.9% in a previously published systematic review on the topic.^8^ The prior information for the parameter ${\beta_{female}}_{kj,Sev=s}$, which allows the incidence to vary by sex was in the form of a non-informative prior (*N*(0,0.01)). We constrained this last prior distribution (T(-0.5,0.1)) to limit how much higher the female incidence can be than the male incidence. The latter is supported by the fact that most TBI and rTBI epidemiological studies demonstrates a higher risk of TBI in males.^4,8–10^ We conducted numerous sensitivity analyses that vary these priors within a reasonable range to ensure that our results were robust.

These constraints we used on the accuracy parameters are shown below:

General accuracy parameter:

$P\left( T_{iprj,Sev=s}=1 \right|L_{ikrj,Sev=s}) \sim uniform(0,1)$

Specific accuracy parameters with constraints are:

$$P\left( T_{i2rj,Sev=2}=1 \right|L_{i2rj,Sev=2})\sim uniform(0.1,1)$$

$$P\left( T_{i3rj,Sev=2}=1 \right|L_{i2rj,Sev=2})\sim uniform(0,0.1)$$

$$P\left( T_{iprj,Sev=2,3}=1 \right|L_{i1rj,Sev=2,3})\sim uniform(0,0.2)$$

Incidence parameters with constraints are:

$$inv.logit\left( {\alpha_{male}}_{krj,Sev=s} \right)\sim uniform(0,0.1)$$

$${\beta_{female}}_{krj,Sev=s}\sim Normal\left( 0,0.01 \right)T(-0.5,0.1)$$

Other parameters derived from the aforementioned parameters:

*Probability of being in a specific class given a case definition response pattern:*

$$P\left( L_{ikrj,Sev=s}=K_{rj,Sev=s}| T_{irj,Sev=s} \right)=\frac{P\left( L_{ikrj,Sev=s} \right)\prod_{pr=1}^{Pr} P\left( T_{iprj,Sev=s} \right|L_{ikrj,Sev=s})}{\sum_{kr=1}^{Kr} P\left( L_{ikrj,Sev=s} \right)\prod_{pr=1}^{Pr} P\left( T_{iprj,Sev=s} \right|L_{ikjr,Sev=s})}$$

*Class-specific incidence across both sexes:*

$$Incidence of Class k_{rj,Sev=s}= \frac{\sum_{i=1}^{32} P\left( L_{ikrj,Sev=s}=K_{rj,Sev=s} \right|T_{irj,Sev=s})*n_{irj,Sev=s}}{\sum_{i=1}^{32} n_{irj,Sev=s}}$$

$${Sensitivity}_{prj,Sev=s}= P\left( T_{iprj,Sev=s}=1 \right|L_{i2rj,Sev=s})$$

${Specificity}_{prj,Sev=s}= P\left( T_{iprj,Sev=s}=0 \right|L_{i1rj,Sev=s})$

$${PPV}_{prj,Sev=s}= \frac{{Sensitivity}_{prj,Sev=s}*{Inc}_{2rj,Sev=s}}{{Sensitivity}_{prj,Sev=s}*{Inc}_{2rj,Sev=s}+(1- {Specificity}_{prj,Sev=s})(1-{Inc}_{2rj,Sev=s})}$$

$${NPV}_{prj,Sev=s}= \frac{{Specificity}_{prj,Sev=s}*{(1-Inc}_{2rj,Sev=s})}{(1-{Sensitivity}_{prj,Sev=s})*{Inc}_{2rj,Sev=s}+({Specificity}_{prj,Sev=s})(1-{Inc}_{2rj,Sev=s})}$$

All parameters above had their *j*=1000 posterior distributions pooled together to ascertain final estimates that are reported.

As mentioned above, the 1000 predicted cohort sizes for each of the 6 cohorts across the 2 incident TBI severity groups and 3 age groups was ascertained in our model. Using these distributions of cohort sizes, we provided summary estimates of the aforementioned parameters by weighting the parameters based on the cohort size of each stratified group. As such, we were able to provide summary estimates across both severity groups of the sensitivity, specificity, PPV, NPV, and rTBI incidence for each age group. We also estimated the overall rTBI incidence across the entire population through the same weighting strategy based on predicted cohort sizes.

$$Parameter across both incident severity groups= \frac{N_{rT,Sev=2}*{parameter}+ N_{rT,Sev=3}*{parameter}}{N_{rT,Sev=2}+N_{rT,Sev=3}}$$

$$N_{rT,Sev=s}= \sum_{j=1}^{1000} N_{rj,Sev=s}$$

Prior information used in sensitivity analyses:

The five following sensitivity analyses were conducted by varying the prior information used on the accuracy parameters that did not use non-informative prior distributions in the main analysis. All other prior distributions defined above were kept the same in the sensitivity analyses, unless otherwise noted below.

*Sensitivity analysis 1:* The prior information on the sensitivity of the discharge abstract database case definition is loosened to a maximum of 25%.

$$P\left( T_{i2rj,Sev=2}=1 \right|L_{i2rj,Sev=2})\sim uniform(0.1,1)$$

$$P\left( T_{i3rj,Sev=2}=1 \right|L_{i2rj,Sev=2})\sim uniform(0,0.25)$$

$$P\left( T_{i3rj,Sev=3}=1 \right|L_{i2rj,Sev=3})\sim uniform(0,0.25)$$

With the other constraints maintained as previously described:

$P\left( T_{iprj,Sev=s}=1 \right|L_{i2rj,Sev=s}) \sim uniform(0,1)$

$$P\left( T_{iprj,Sev=2,3}=1 \right|L_{i1rj,Sev=2,3})\sim uniform(0,0.2)$$

$$inv.logit\left( {\alpha_{male}}_{2j,Sev=s} \right)\sim uniform(0,0.1)$$

$${\beta_{female}}_{2j,Sev=s}\sim Normal\left( 0,0.01 \right)T(-0.5,0.1)$$

*Sensitivity analysis 2:* The specificity of the radiological examination case definition is forced to be less than 95%.

$$P\left( T_{i5rj,Sev=2,3}=1 \right|L_{i1rj,Sev=2,3})\sim uniform(0.05,0.2)$$

With the other constraints maintained as previously described:

$P\left( T_{iprj,Sev=s}=1 \right|L_{i2rj,Sev=s}) \sim uniform(0,1)$

$$P\left( T_{i2rj,Sev=2}=1 \right|L_{i2rj,Sev=2})\sim uniform(0.1,1)$$

$$P\left( T_{i3rj,Sev=2}=1 \right|L_{i2rj,Sev=2})\sim uniform(0,0.1)$$

$$P\left( T_{iprj,Sev=2,3}=1 \right|L_{i1rj,Sev=2,3})\sim uniform(0,0.2)$$

$$inv.logit\left( {\alpha_{male}}_{2j,Sev=s} \right)\sim uniform(0,0.1)$$

$${\beta_{female}}_{2j,Sev=s}\sim Normal\left( 0,0.01 \right)T(-0.5,0.1)$$

*Sensitivity analysis 3:* The specificity of the outpatient physician claim case definition is forced to be less than 95%.

$$P\left( T_{i1rj,Sev=2,3}=1 \right|L_{i1rj,Sev=2,3})\sim uniform(0.05,0.2)$$

With the other constraints maintained as previously described:

$P\left( T_{iprj,Sev=s}=1 \right|L_{i2rj,Sev=s}) \sim uniform(0,1)$

$$P\left( T_{i2rj,Sev=2}=1 \right|L_{i2rj,Sev=2})\sim uniform(0.1,1)$$

$$P\left( T_{i3rj,Sev=2}=1 \right|L_{i2rj,Sev=2})\sim uniform(0,0.1)$$

$$P\left( T_{iprj,Sev=2,3}=1 \right|L_{i1rj,Sev=2,3})\sim uniform(0,0.2)$$

$$inv.logit\left( {\alpha_{male}}_{2j,Sev=s} \right)\sim uniform(0,0.1)$$

$${\beta_{female}}_{2j,Sev=s}\sim Normal\left( 0,0.01 \right)T(-0.5,0.1)$$

*Sensitivity analysis 4:* The specificity of the emergency physician claims and the discharge abstract database case definitions are not constrained.

$$P\left( T_{i2rj,Sev=2,3}=1 \right|L_{i1rj,Sev=2})\sim uniform(0,1)$$

$$P\left( T_{i3rj,Sev=2,3}=1 \right|L_{i1rj,Sev=2})\sim uniform(0,1)$$

With the other constraints maintained as previously described:

$P\left( T_{iprj,Sev=2,3}=1 \right|L_{i2rj,Sev=2,3}) \sim uniform(0,1)$

$$P\left( T_{i2rj,Sev=2}=1 \right|L_{i2rj,Sev=2})\sim uniform(0.1,1)$$

$$P\left( T_{i3rj,Sev=2}=1 \right|L_{i2rj,Sev=2})\sim uniform(0,0.1)$$

$$P\left( T_{iprj,Sev=2,3}=1 \right|L_{i1rj,Sev=2,3})\sim uniform(0,0.2)$$

$$inv.logit\left( {\alpha_{male}}_{2j,Sev=s} \right)\sim uniform(0,0.1)$$

$${\beta_{female}}_{2j,Sev=s}\sim Normal\left( 0,0.01 \right)T(-0.5,0.1)$$

*Sensitivity analysis 5:* The incidence for males is constrained to being less than 15% instead of 10%.

$$inv.logit\left( {\alpha_{male}}_{2j,Sev=2,3} \right)\sim uniform(0,0.15)$$

With the other constraints maintained as previously described:

$P\left( T_{iprj,Sev=s}=1 \right|L_{ikrj,Sev=s}) \sim uniform(0,1)$

$$P\left( T_{i2rj,Sev=2}=1 \right|L_{i2rj,Sev=2})\sim uniform(0.1,1)$$

$$P\left( T_{i3rj,Sev=2}=1 \right|L_{i2rj,Sev=2})\sim uniform(0,0.1)$$

$$P\left( T_{iprj,Sev=2,3}=1 \right|L_{i1rj,Sev=2,3})\sim uniform(0,0.2)$$

$${\beta_{female}}_{2j,Sev=s}\sim Normal\left( 0,0.01 \right)T(-0.5,0.1)$$

**eAppendix 3: Posterior predictive distribution (Bayesian *p*-values) to assess model fit by age group**

Typical approaches to assessing model fit in other statistical models, such as Discrepancy ($\chi$^2^ statistic and the likelihood ratio), are not appropriate to assess model fit in latent class models.^11^ For latent class analysis, conducting posterior predictive checks that compare the observed and predicted agreement between pairs of tests (or case definitions), $pq,$ has been shown to be an adequate way of assessing model fit and ensuring there is no residual correlation between pairs of tests.^12,13^ We drew 3000 samples from the posterior predictive distribution for each of *j*=1000 simulated cohorts to establish the “*expected”* counts, ${n.new}_{irj},$ of each of the 16 case definition response patterns for each sex (a total of $T_{irj}$ = 32 response patterns).^11^ We also used the *j*=1000 simulated cohorts for each of the case definition response patterns,$n_{irj,Sev=s}$, which was established using predictions of each response patterns from our previous analysis on incident TBI, as the *“observed”* counts. The observed and expected agreement between pairs of tests, ($pq)$, was estimated as detailed below. We then estimated the probability that the observed agreement would be greater than the predicted agreement within the 3000 samples that were drawn for each pair of tests (${P(Observed agreement}_{pqrj,Sev=s}$) >$P({Predicted agreement}_{pqrj,Sev=s}))$, which is also known as a Bayesian *p*-value, and across the *j*=1000 simulated cohorts.^13^ When these probabilities are close to 0 or 1 there is evidence to suggest that model fit may be inappropriate. We conducted this analysis for each age group across the “mildest/more severe” and “most severe” incident TBI cohorts across the *j*=1000 simulated cohorts. The “*r*” subscript indicates that the parameter in the model represents the rTBI analysis, in contrast to the incident TBI analysis from our previously completed investigation on the topic.

When these probabilities are very close to 0 or 1, there may be evidence that model fit is inappropriate.

$${Predicted agreement}_{pqrj,Sev=s}=\frac{\sum_{i=1}^{64} {n.new}_{irj}*(T_{iprj,Sev=s}T_{iqrj,Sev=s}+\left( 1-T_{iprj,Sev=s} \right)\left( 1-T_{iqrj,Sev=s} \right))}{\sum_{i=1}^{64} {n.new}_{irj,Sev=s}}$$

$${Observed agreement}_{pqrj,Sev=s}=\frac{\sum_{i=1}^{64} n_{irj,Sev=s}*(T_{iprj,Sev=s}T_{iqrj,Sev=s}+\left( 1-T_{iprj,Sev=s} \right)\left( 1-T_{iqrj,Sev=s} \right))}{\sum_{i=1}^{64} n_{irj,Sev=s}}$$

The *j*=1000 simulations were combined together to obtain the Bayesian *p*-value of each pair of case definitions for each age group and index TBI severity cohort.

**eAppendix 4: Crude and adjusted median time to rTBI recurrence by age group**

The crude median time to rTBI was estimated for each age group using the earliest case definition that an individual was positive for after their incident TBI. However, an adjusted median time to recurrence was also assessed to assess the validity of this crude measure. To do the latter, we estimated the probability that an individual had an incident TBI based on their case definition response pattern for incident TBI. We also estimated the same probability for rTBI using the case definition response pattern for rTBI. The product of these probabilities was the overall probability that the individual was a true rTBI case. The crude recurrence time was defined as the earliest time when a patient met a case definition for rTBI during the 1-year follow-up period. These probabilities were used as weights for the crude time to recurrence estimate mentioned above. *j=*1000 simulations of the accuracy parameters from the incident TBI and rTBI analyses were taken to conduct 1000 analyses, such that the uncertainty in the accuracy parameters was maintained in this analysis. 1000 estimates for median time to rTBI were established for each age group. The median of these 1000 analyses represented the overall median time to recurrence, as shown below. The “*r*” subscript indicates that the parameter in the model represents the rTBI analysis, in contrast to the incident TBI analysis from our previously completed investigation on the topic.

The probability of incident TBI and rTBI is as follows:

*For incident TBI:*

$${P\left( L_{ikj}=K_{j}| T_{ij} \right)}={{P\left( L_{ikj} \right)}\prod_{p=1}^{P} P\left( T_{ipj} \right|L_{ikj})}$$

*For rTBI:*

$${P\left( L_{ikr}=K_{rj}| T_{irj} \right)}={P\left( L_{ikrj} \right)}\prod_{pr=1}^{Pr} {P\left( T_{iprj} \right|L_{ikrj})}$$

Where K is an incident case, Kr is recurrent case, $L_{ik}$/$L_{ikr}$ is the incidence across all classes for incident TBI/rTBI, $p$ is 1 of 5 case definitions, $pr$ is1 of 4 case definitions, $T_{i}$ is 1 of 32, and $T_{ir}$ is 1/16 case definition response patterns for the *i*^th^ individual with suspected rTBI.

The accuracy parameters (as distributions), across all severities of incident TBI, $P\left( T_{ip} \right|L_{ik})$ and $P\left( T_{ipr} \right|L_{ikr})$ were established from the previously completed study on incident TBI and the main analysis of the current study on rTBI, respectively.

${Weight}_{ij,Sev=s}=$ ${P\left( L_{ikj}=K_{j}| T_{i} \right)}*{P\left( L_{ikrj}=K_{rj}| T_{irj} \right)}$

$${Adjusted time to recurrence}_{j}=\frac{{rec_{time}}_{i}*{Weight}_{ij}}{\sum_{i=1}^{n} {Weight}_{ij}}$$

${Median time to recurrence}={Median (adjusted time to recurrence}_{j)}$ for *j*=1:1000

Where *n* is the total number of patients with *suspected* rTBI in each age group cohort.

**eAppendix 5: Approach to using parameter estimates from study to adjust for measurement error in administrative health data**

The parameter estimates provided by this study can be used by other investigators such that measurement-error adjusted rTBI incidence estimate can be estimated in their own population. The sensitivity and specificity parameter of the case definition that is being used by the investigator is all that is needed to make the adjustment. Any case definition that we assessed in our study can be used to make the measurement error adjustment. As such, this approach is quite flexible since investigators in different jurisdictions may have access only to certain case definitions and not necessarily the five case definitions we investigated.

By using this approach, the investigator is assuming that heath care utilization patterns in their own population is similar to our study and that the epidemiology of rTBI is also similar. These are reasonable assumptions since many studies have shown that the epidemiology of TBI is quite homogeneous across developed countries.^14^ However, if the assumptions are deemed not to be reasonable, a similar approach to what we completed in this study would need to be adopted to adjust for the measurement error.

Below, we describe how the adjustment can be made using the sensitivity and specificity parameter of a given case definition. A standard 2x2 table used to interpret the performance of a diagnostic test can be used to facilitate the understanding of the calculation.^5^

|  | **True diagnosis +** | **True diagnosis -** | **Total** |
| --- | --- | --- | --- |
| **Case definition +** | Y | a-Y | a |
| **Case definition -** | X | b-X | b |
| Total | Y+X | a+b-Y-X | a+b |

a = total number of individuals positive for a given case definition (observed)

b = total number of individuals negative for a given case definition (observed)

$$Sensitivity=\frac{Y}{Y+X}$$

$$Specificity=\frac{b-X}{a+b-Y-X}$$

As such, we have 2 equations to solve and 2 unknown variables (Y and X). Using simple algebra, Y and X are calculated.

Then the adjusted incidence is calculated as:

$$Adjusted incidence= \frac{Y+X}{a+b}$$

**eTable 1: Model fit assessment using Bayesian *p-*values across each age group in the main analysis**

|  | **Children** | | **Adults** | | **Elderly** | |
| --- | --- | --- | --- | --- | --- | --- |
|  | Pr (Observed > Predicted) | | | | | |
|  |  | | | | | |
| **Case definition pair (*pq*)** | Mildest/more severe | Most severe | Mildest/more severe | Most severe | Mildest/more severe | Most severe |
| **1,2** | 0.30 | 0.55 | 0.60 | 0.55 | 0.57 | 0.29 |
| **1,3** | 0.27 | 0.65 | 0.44 | 0.6 | 0.11 | 0.40 |
| **1,4** | 0.61 | 0.53 | 0.44 | 0.35 | 0.46 | 0.28 |
| **2,3** | 0.68 | 0.53 | 0.50 | 0.18 | 0.84 | 0.14 |
| **2,4** | 0.44 | 0.60 | 0.50 | 0.38 | 0.47 | 0.35 |
| **3,4** | 0.69 | 0.63 | 0.63 | 0.56 | 0.83 | 0.78 |

Model fit assessing the observed and predicted agreement between pairs of case of definitions pooled across both severities of index TBI. The probability that the observed agreement is greater than the predicted agreement between pairs of case definitions is used to assess whether or not model fit is appropriate.^11^ When probabilities (also known as Bayesian *p*-values) are close to 0 or 1, then model fit may be inappropriate (eAppendix 3). The pairs of case definitions are as defined in eAppendix 2. 1 = outpatient physician claim, 2 = emergency department physician claim, 3 = discharge abstract database, 4 = radiological examination of the head in the context of any trauma diagnosis.

**eTable 2: Distribution of rTBI cases by case definition response patterns and by age group**

| Case definition response patterns | | | |  |  |  |
| --- | --- | --- | --- | --- | --- | --- |
| Outpatient  claim | Emergency department  claim | Discharge abstract  database | Radiological examination of the head | Children | Adults | Elderly |
| - | - | - | - | 28876 | 35281 | 22121 |
| - | - | - | + | 100 | 544 | 1292 |
| - | - | + | - | 22 | 253 | 306 |
| - | - | + | + | 11 | 115 | 208 |
| - | + | - | - | 723 | 333 | 139 |
| - | + | - | + | 58 | 220 | 331 |
| - | + | + | - | 14 | 7 | 11 |
| - | + | + | + | 18 | 55 | 110 |
| + | - | - | - | 516 | 1425 | 261 |
| + | - | - | + | 48 | 91 | 38 |
| + | - | + | - | 1 | 30 | 11 |
| + | - | + | + | 4 | 36 | 24 |
| + | + | - | - | 40 | 36 | 3 |
| + | + | - | + | 9 | 47 | 11 |
| + | + | + | - | 1 | 1 | 0 |
| + | + | + | + | 2 | 12 | 15 |
| Total | | | | 30443 | 38486 | 24881 |

There are a total of 16 response patterns possible given that 4 case definitions were used for the Bayesian latent class analysis.

**eTable 3: Adjusted median time to recurrence stratified by incident TBI severity**

|  | Median time to rTBI (days) (95% CrI) | |
| --- | --- | --- |
|  | “Mildest and more severe”  incident TBI cohort | “Most severe”  incident TBI cohort |
| Children (0-17 years) | 117.38 (110.84 , 125.41) | 99.10 (92.66 , 108.20) |
| Adults (18-64 years) | 74.07 (71.44 , 76.99) | 57.13 (53.91 , 63.03) |
| Elderly (>=65 years) | 106.31 (103.67 , 109.17) | 86.26 (79.76 , 93.92) |

In the main analysis, the median time to recurrence across both index TBI severities is reported. Here, we conducted the same analysis as explained in eAppendix 4 but did stratified assessments based on index TBI severity.

**eTable 4: Sensitivity analysis assessing crude rTBI incidence depending on time period when suspected rTBI are excluded**

|  | **>7 days** | **>30 days** |
| --- | --- | --- |
| *Children*  Suspected incident TBI cases  Suspected rTBI cases  Crude incidence (per 100 person-years) | 30433  1567  5.14 | 30433  1178  3.87 |
| *Adults*  Suspected incident TBI cases  Suspected rTBI cases  Crude incidence (per 100 person-years | 38486  3205  8.33 | 38486  1762  4.58 |
| *Elderly*  Suspected incident TBI cases  Suspected rTBI cases  Crude incidence (per 100 person-years | 24881  2760  11.09 | 24881  2271  9.12 |

In our main analysis, we assumed that all suspected TBI claims within the first 7 days after an incident TBI were related to the first TBI and not a rTBI. We conducted a sensitivity analysis where we extended the period where we excluded TBI claims for 30 days after the incident injury.

**eFigure 1: Conceptual diagrams demonstrating the latent classes and the observed variables used in the incident TBI and rTBI analyses**

**eFigure 1A:**

This conceptual diagram outlines the incident TBI latent class analysis that was previously completed.^4^ This model was used to predict 1000 cohorts of incident TBI patients across the three age groups in the main analysis. The incident cohorts were predicted into two severity classes: “mildest/more severe” and “most severe” incident TBI patients.

**eFigure 1B:**

This conceptual diagram demonstrates the two latent classes used in the present rTBI analysis. The predicted incident TBI cohorts, described in the previous heuristic diagram, were used to complete this analysis.

**eFigure 2: Sensitivity analyses**

Five sensitivity analyses were completed that varied the prior information we used the main analysis within plausible ranges. The estimates from these five analyses were compared to the estimates from the main analysis above. The prior information varied in these three sensitivity analyses is as described at the end of eAppendix 2. “Outpatient” = outpatient claims, “ER” = emergency room claims, “DAD” = discharge abstract database, “Radiology” = radiological examination claim in the context of any trauma diagnosis.

**References:**

1. Taylor CA, Bell JM, Breiding MJ, Xu L. Traumatic Brain Injury-Related Emergency Department Visits, Hospitalizations, and Deaths - United States, 2007 and 2013. *Morb Mortal Wkly Rep Surveill Summ Wash DC 2002*. 2017;66(9):1-16. doi:10.15585/mmwr.ss6609a1

2. St Germaine-Smith C, Metcalfe A, Pringsheim T, et al. Recommendations for optimal ICD codes to study neurologic conditions A systematic review. *Neurology*. 2012;79(10):1049-1055.

3. Mission | RAMQ. Published 2016. Accessed October 16, 2016. http://www.ramq.gouv.qc.ca/en/regie/Pages/mission.aspx

4. Lasry O, Dendukuri N, Marcoux J, Buckeridge DL. Accuracy of Administrative Health Data for Surveillance of Traumatic Brain Injury: A Bayesian Latent Class Analysis. *Epidemiology*. 2018;29(6):876. doi:10.1097/EDE.0000000000000888

5. Joseph L, Gyorkos TW, Coupal L. Bayesian estimation of disease prevalence and the parameters of diagnostic tests in the absence of a gold standard. *Am J Epidemiol*. 1995;141(3):263-272.

6. Jasra A, Holmes CC, Stephens DA. Markov Chain Monte Carlo Methods and the Label Switching Problem in Bayesian Mixture Modeling. *Stat Sci*. 2005;20(1):50-67. doi:10.1214/088342305000000016

7. Swaine BR, Tremblay C, Platt RW, Grimard G, Zhang X, Pless IB. Previous head injury is a risk factor for subsequent head injury in children: a longitudinal cohort study. *Pediatrics*. 2007;119(4):749-758.

8. Lasry O, Liu EY, Powell GA, Ruel-Laliberté J, Marcoux J, Buckeridge DL. Epidemiology of recurrent traumatic brain injury in the general population: A systematic review. *Neurology*. 2017;89(21):2198-2209. doi:10.1212/WNL.0000000000004671

9. Theadom A, Parmar P, Jones K, et al. Frequency and impact of recurrent traumatic brain injury in a population-based sample. *J Neurotrauma*. 2015;32(10):674-681.

10. Feigin VL, Theadom A, Barker-Collo S, et al. Incidence of traumatic brain injury in New Zealand: a population-based study. *Lancet Neurol*. 2013;12(1):53-64. doi:10.1016/S1474-4422(12)70262-4

11. Garrett ES, Zeger SL. Latent class model diagnosis. *Biometrics*. 2000;56(4):1055-1067.

12. Dendukuri N, Hadgu A, Wang L. Modeling conditional dependence between diagnostic tests: A multiple latent variable model. *Stat Med*. 2009;28(3):441-461. doi:10.1002/sim.3470

13. Gelman A, Meng X, Stern H. Posterior Predictive Assessment of Model Fitness Via Realized Discrepancies. *Stat Sin*. 1996;6:733-807.

14. James SL, Theadom A, Ellenbogen RG, et al. Global, regional, and national burden of traumatic brain injury and spinal cord injury, 1990–2016: a systematic analysis for the Global Burden of Disease Study 2016. *Lancet Neurol*. 2019;18(1):56-87. doi:10.1016/S1474-4422(18)30415-0
